# Chelidonium majus-Induced Hepatitis: A Case Report of Herb-Induced Liver Injury

**DOI:** 10.7759/cureus.109572

**Published:** 2026-05-24

**Authors:** Nikos Adamidis, Sofia Adamidi, Vasiliki E Georgakopoulou, Sofia Miliou, Argyroula Karampela, Epameinondas Stratopoulos, Theodora Margariti, Georgios Sakizlis, Sotirios Adamidis

**Affiliations:** 1 First Department of Internal Medicine, Sismanogleio General Hospital, Athens, GRC; 2 Department of Internal Medicine, Charlton Memorial Hospital, Massachusetts, USA; 3 Department of Pathophysiology, Laiko General Hospital, Athens, GRC; 4 First Department of Internal Medicine, Athens Medical Center, Athens, GRC; 5 School of Medicine, National and Kapodistrian University of Athens, Athens, GRC; 6 Department of Endocrine Surgery, Athens Medical Center, Athens, GRC; 7 Department of Endocrine Surgery, Helios Universitätsklinikum Wuppertal, Wuppertal, DEU; 8 Department of Gastroenterology, Athens Medical Center, Athens, GRC; 9 First Department of Internal Medicine, Athens Medical Group, Athens, GRC

**Keywords:** acute hepatitis, chelidonium majus, drug-induced liver injury, greater celandine, herb-induced liver injury

## Abstract

Herb-induced liver injury can present with nonspecific symptoms and may be difficult to recognize when herbal exposure is not initially disclosed. A 60-year-old female patient presented with jaundice and scleral icterus, nausea, anorexia, and fatigue after recent initiation of a herbal preparation identified as *Chelidonium majus*,* *commonly known as greater celandine. Clinical examination revealed icterus. Initial laboratory testing showed aspartate aminotransferase, alanine aminotransferase, total bilirubin, direct bilirubin, and international normalized ratio at pathologically high levels. Viral hepatitis was excluded. Overall, the findings of the autoimmune evaluation did not support a definitive diagnosis of autoimmune hepatitis. Magnetic resonance imaging/magnetic resonance cholangiopancreatography and abdominal ultrasonography showed no biliary obstruction or focal hepatic lesion. Liver biopsy demonstrated an acute hepatitis-like pattern with portal inflammation, ductular reaction, centrilobular necrosis, apoptotic hepatocytes, ceroid-laden macrophages, giant-cell hepatocyte transformation, and mild non-bridging fibrosis. The suspected herbal product was discontinued, and the patient received supportive treatment with progressive biochemical improvement. This case supports the need to consider herbal preparations in the diagnostic evaluation of acute hepatitis, particularly when common viral, autoimmune, and obstructive causes have been excluded.

## Introduction

Drug-induced liver injury (DILI) remains one of the most challenging diagnostic entities in clinical hepatology because of its heterogeneous clinical presentation, unpredictable course, and absence of a single confirmatory biomarker. Its spectrum ranges from asymptomatic abnormalities in liver biochemistry to clinically apparent hepatitis, cholestatic jaundice, acute liver failure, or persistent liver injury [1-3]. Current international recommendations emphasize that the diagnosis of DILI requires an integrated assessment of the temporal relationship between exposure and liver injury, the biochemical pattern of damage, the exclusion of alternative etiologies, and the clinical course after withdrawal of the suspected agent [1-3]. This approach is especially important in idiosyncratic DILI, which is typically not dose-dependent and may occur only in susceptible individuals exposed to drugs, herbal products, or dietary supplements that are otherwise tolerated by most users [1-3].

The increasing use of complementary and alternative medicines has broadened the clinical spectrum of DILI to include herb-induced liver injury (HILI). Herbal preparations and dietary supplements are frequently consumed without medical supervision and may contain multiple biologically active compounds. Moreover, patients often perceive these products as safe because they are “natural,” and may not report their use unless specifically questioned [1-3]. This can delay diagnosis and obscure the true cause of liver injury. Causality assessment is further complicated by variability in plant species, preparation methods, doses, exposure durations, contamination, adulteration, and product mislabeling. Therefore, HILI should be considered in patients with otherwise unexplained acute hepatitis, particularly when viral, autoimmune, metabolic, and obstructive causes have been excluded [1-3].

Structured causality assessment is central to the evaluation of suspected DILI and HILI. The updated Roussel Uclaf Causality Assessment Method (RUCAM) provides a liver-specific framework for assessing suspected DILI and HILI [4]. It evaluates key domains, including time to onset from the start of exposure or cessation, clinical course after withdrawal of the suspected agent, risk factors, concomitant drugs or herbal products, exclusion of alternative causes, prior information on hepatotoxicity, and, when available, response to unintentional re-exposure [4]. In the context of herbal hepatotoxicity, RUCAM is particularly useful because causality cannot be inferred from temporal association alone. Instead, the diagnosis requires a systematic and transparent evaluation of competing etiologies and the clinical evolution after discontinuation of the suspected product [4].

In DILI/HILI evaluation, the biochemical pattern of liver injury is commonly classified using the R-ratio, calculated as (serum alanine aminotransferase (ALT) divided by its upper limit of normal) divided by (serum alkaline phosphatase (ALP) divided by its upper limit of normal). An R-ratio ≥5 indicates a hepatocellular pattern, ≤2 indicates a cholestatic pattern, and values between 2 and 5 indicate a mixed pattern [1-4]. This classification is clinically useful because *Chelidonium majus*-induced liver injury has most often been reported as an acute hepatitis-like presentation, frequently with jaundice and marked aminotransferase elevation [5-10]. *Chelidonium majus, *commonly known as greater celandine, contains several biologically active alkaloids, including chelidonine, chelerythrine, sanguinarine, berberine, and coptisine, which have been proposed to contribute to its pharmacological and toxicological effects [5].

*Chelidonium majus *is a plant of the Papaveraceae family traditionally used in European herbal medicine, particularly for gastrointestinal, biliary, and hepatobiliary complaints. Despite this traditional use, oral preparations of *Chelidonium majus* have been repeatedly associated with acute liver injury. Previous reports, including both older case series and more recent case reports, have shown that *Chelidonium majus*-induced liver injury may present with a hepatitic biochemical pattern, jaundice, and symptoms that can mimic acute viral or autoimmune hepatitis [5-10]. These data support the clinical relevance of *Chelidonium majus* as a potential cause of HILI and highlight the need to consider herbal exposure in patients with otherwise unexplained acute hepatitis.

From a clinical perspective, *Chelidonium majus*-induced liver injury is important for several reasons. First, it challenges the assumption that herbal products used for hepatobiliary symptoms are inherently hepatoprotective or harmless. Second, its presentation may resemble other common causes of acute hepatitis, potentially leading to extensive investigations and diagnostic delay. Third, because no specific antidote exists, early recognition and prompt discontinuation of the suspected herbal preparation remain the cornerstone of management [1-3]. In patients with jaundice, coagulopathy, encephalopathy, or progressive biochemical deterioration, close monitoring is required, and referral to a specialized liver unit should be considered according to the severity of liver dysfunction [1-3].

We report the case of a 60-year-old female patient who developed jaundice, nausea, anorexia, and fatigue after the recent initiation of *Chelidonium majus*. Viral hepatitis, autoimmune hepatitis, and obstructive hepatobiliary disease were excluded, while liver biopsy showed findings compatible with an acute hepatitis-like toxic injury. This case adds to the existing literature on *Chelidonium majus*-induced liver injury. It underscores the importance of obtaining a detailed history of herbal and dietary supplement use in every patient presenting with unexplained acute hepatitis.

## Case presentation

A 60-year-old female patient presented with complaints of yellow discoloration of the skin and sclerae, nausea, anorexia, and fatigue. Her past medical history was notable for psoriasis, for which she was not receiving systemic treatment, and dyslipidemia. She had recently started taking a herbal preparation identified as *Chelidonium majus*. The patient reported that she had started taking an oral herbal preparation containing *Chelidonium majus* approximately three weeks before the onset of symptoms. The product was purchased over the counter as a liquid herbal extract and was taken for dyspeptic and hepatobiliary symptoms. According to the patient’s recollection, she consumed approximately 20-25 drops, diluted in water, twice daily, which corresponds to the manufacturer’s recommended dose. However, the exact alkaloid concentration and full product composition could not be verified. She denied use of other herbal products, weight-loss supplements, or newly introduced prescription medications during the same period. The latency period of approximately 21 days was considered compatible with idiosyncratic HILI and was incorporated into the RUCAM causality assessment. No other clearly documented hepatotoxic drug exposure was identified in the available medical records.

On clinical examination, the patient was icteric, with evident jaundice of the skin and sclerae. The available clinical documentation did not describe signs of chronic liver disease, abdominal distension, clinically evident ascites, or overt hepatic encephalopathy at presentation. Given the presence of jaundice and systemic symptoms, laboratory evaluation was performed to assess the severity and pattern of liver injury.

Initial laboratory testing demonstrated severe acute liver injury with marked hyperbilirubinemia, predominantly hepatocellular enzyme elevation, and impaired coagulation parameters, as summarized in Table 1.

**Table 1:** Initial laboratory findings at presentation Anti-HBc: antibody to hepatitis B core antigen, HBeAg: hepatitis B e antigen, anti-HEV: antibody to hepatitis E virus

Laboratory parameter	Patient value	Reference range	Unit
Total bilirubin	12.98	<1.2	mg/dL
Direct bilirubin	9.33	<0.50	mg/dL
Aspartate aminotransferase	1579	11-34	U/L
Alanine aminotransferase	1444	<34	U/L
Gamma-glutamyl transferase	329	<38	U/L
Alkaline phosphatase	206	35-104	U/L
Prothrombin time	20.7	9.7-13.5	seconds
International normalized ratio	1.64	0.80-1.20	-
White blood cells	7.85	4.00-11.00	×10³/μL
Hemoglobin	13.9	12.0-16.0	g/dL
Platelets	160	150-400	×10³/μL
Antinuclear antibodies	Positive, 1:160	Negative	titer
Anti-mitochondrial antibodies	Negative	Negative	-
Anti-smooth muscle antibodies	Negative	Negative	-
Liver cytosol antibodies	Negative	Negative	-
Immunoglobulin G	1770	700-1600	mg/dL
Immunoglobulin M	85	45-230	mg/dL
Anti-HBc immunoglobulin M	Negative	Negative	-
HBeAg	Negative	Negative	-
Anti-HEV immunoglobulin M	Negative	Negative	-

A broad etiological investigation was subsequently undertaken. Viral hepatitis was excluded, with negative testing for hepatitis B, C, A, and D viruses according to the histopathology report. Additional serological testing showed negative anti-HBc immunoglobulin M, HBeAg, and anti-HEV immunoglobulin M. Autoimmune evaluation showed antinuclear antibody positivity at a titer of 1:160, while anti-mitochondrial, anti-smooth muscle, and liver cytosol antibodies were negative. Serum immunoglobulin G was moderately elevated (1770 mg/dL), whereas immunoglobulin M was within the normal range. Overall, the available clinical and serological findings did not support a definite diagnosis of autoimmune hepatitis.

During the initial days of hospitalization, abdominal imaging was performed to exclude obstructive or structural hepatobiliary disease. Magnetic resonance imaging and magnetic resonance cholangiopancreatography showed a liver of normal size and morphology, without focal suspicious hepatic lesions or evidence of fatty infiltration. There was no intrahepatic or extrahepatic bile duct dilatation, and no gallstones or obstructive biliary process was identified. Mildly increased T2 signal intensity in the hepatic parenchyma, with subtle periportal edema, was described as a nonspecific finding, possibly related to an inflammatory process. The portal vein had a normal diameter and no filling defects. A small cystic lesion measuring approximately 8 mm was also noted in the uncinate process of the pancreas. It was interpreted as a possible intraductal papillary mucinous neoplasm or a serous cystic adenoma, with no suspicious imaging features.

On the second day, abdominal ultrasonography similarly showed a liver of normal size and echotexture, without focal parenchymal lesions or dilatation of the intrahepatic or extrahepatic bile ducts, and with a common bile duct diameter of 0.5 cm. The gallbladder showed wall thickening and stratification up to 0.6 cm, with mild pericholecystic edema, but without clear evidence of gallstones. Portal venous flow was preserved.

On the seventh day, transient elastography was performed because of the severity of the liver injury. The median liver stiffness was 18 kPa (IQR 18%), and the controlled attenuation parameter was 187, consistent with absent steatosis. The report explicitly noted that, because of the high inflammatory activity, reliable staging of fibrosis was not possible at that time. Although the liver stiffness value of 18 kPa was numerically high, it was interpreted with caution because severe acute hepatic inflammation can transiently increase liver stiffness, leading to an overestimation of fibrosis. Therefore, this value was not considered evidence of established cirrhosis, particularly because the liver biopsy showed preserved architecture and only mild non-bridging fibrosis.

**Figure 1:**
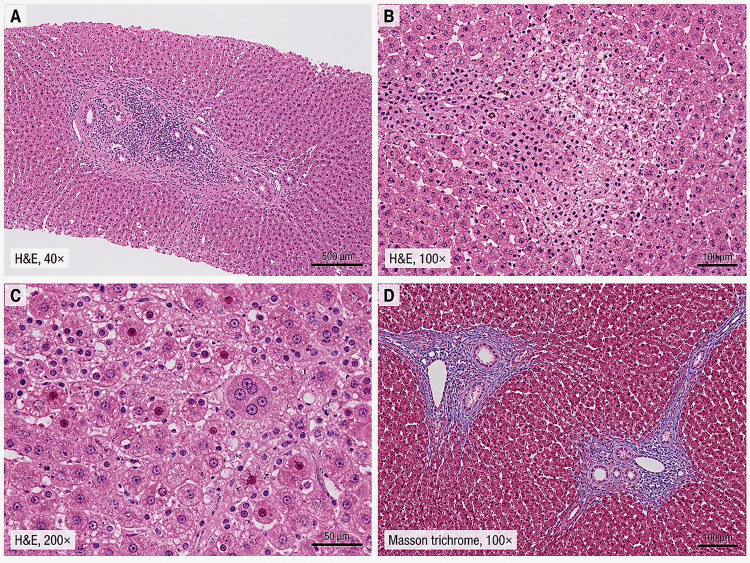
Representative liver biopsy findings consistent with an acute hepatitis-like pattern of DILI or HILI (A) Low-power H&E section showing preserved hepatic architecture with mild-to-moderate chronic inflammatory infiltrates within the portal tract and mild ductular reaction (H&E, ×40). (B) Higher-power H&E section demonstrating lobular necroinflammatory activity, predominantly in the centrilobular region, with focal hepatocyte dropout, scattered inflammatory cells, and ceroid-laden macrophages, compatible with acute hepatitic injury (H&E, ×100). (C) High-power H&E section highlighting hepatocellular injury with numerous apoptotic hepatocytes/acidophil bodies, ballooning degeneration, and multinucleated or giant-cell hepatocyte transformation (H&E, ×200). (D) Masson trichrome staining showing mild portal/periportal fibrosis without bridging fibrosis or established cirrhotic architectural remodeling (Masson trichrome, ×100). H&E: hematoxylin and eosin, DILI: drug-induced liver injury, HILI: herb-induced liver injury

Because the etiology of the liver injury remained unclear and the biochemical abnormalities were severe, a liver biopsy was subsequently performed. Histological examination showed preserved hepatic architecture, with eleven portal tracts available for evaluation. The portal tracts demonstrated mild to moderate chronic inflammation, mild ductular reaction with polymorphonuclear cells, and mild non-bridging fibrosis. There was no significant bile duct-type or lymphocytic duct lesion, and plasma cells were not identified. In the lobules, centrilobular necrosis was present, with numerous apoptotic hepatocytes, acidophil bodies, and ceroid-laden macrophages. Mild reticulin fibrosis was noted in the affected areas, but neutrophilic bridging necrosis was not identified. Hepatocytes showed diffuse giant-cell transformation and focal chronic cholangitis. Masson trichrome staining did not reveal additional significant fibrosis. There was no bilirubinostasis, chronic cholestasis, steatosis, iron deposition, or alpha-1 antitrypsin accumulation. No evidence of malignancy was observed. Representative histopathological findings are shown in Figure 1.

The final histopathological interpretation was a hepatitis-like inflammatory liver disease compatible with acute hepatitis. The pathology report specifically noted that the herbal preparation administered to the patient, *Chelidonium majus*, had been described in the international literature as a cause of acute hepatitis in a limited number of cases and recommended clinicopathological correlation and follow-up.

After discontinuation of the suspected herbal preparation, the patient was managed conservatively with hydration and vitamin supplementation. Because of impaired coagulation, she received vitamin K for three days. Elevated blood ammonia levels were treated with rifaximin and lactulose. During hospitalization, she did not develop major complications.

During follow-up, serial laboratory testing showed gradual biochemical improvement. Total bilirubin decreased to 3.86 mg/dL (reference range: <1.2 mg/dL), direct bilirubin to 2.58 mg/dL (reference range: <0.50 mg/dL), AST to 366 U/L (reference range: 11-34 U/L), ALT to 582 U/L (reference range: <34 U/L), and GGT to 186 U/L (reference range: <38 U/L). Coagulation parameters also improved, with INR decreasing to 1.25 (reference range: 0.80-1.20). The patient was discharged in improved clinical condition.

A structured causality assessment was performed using the updated RUCAM for hepatocellular injury. The initial R-ratio was approximately 21.4, calculated as (ALT/ULN)/(ALP/ULN) = (1444/34)/(206/104), confirming a hepatocellular pattern. The total RUCAM score was 9 points, indicating a highly probable causal relationship between exposure to *Chelidonium majus* and liver injury. The score was assigned as follows: compatible time to onset after initiation of the herbal preparation (+2), decrease in ALT of more than 50% after withdrawal of the suspected agent (+2), age above 55 years (+1), absence of clearly documented concomitant hepatotoxic drugs (0), exclusion of relevant alternative causes including viral hepatitis, obstructive biliary disease, and definite autoimmune hepatitis (+2), previous published evidence of hepatotoxicity associated with *Chelidonium majus* (+2), and absence of rechallenge (0). Rechallenge was not performed and would not have been ethically appropriate.

Based on the recent exposure to *Chelidonium majus*, the marked hepatocellular injury with jaundice, the exclusion of viral and obstructive causes, the absence of convincing evidence for autoimmune hepatitis, the compatible liver biopsy findings, and the improvement after withdrawal of the suspected herbal product, the final diagnosis was considered HILI due to *Chelidonium majus*.

## Discussion

This case describes a probable episode of *Chelidonium majus*-induced liver injury in a 60-year-old female patient who presented with jaundice, nausea, anorexia, and fatigue after recent exposure to *Chelidonium majus*. The diagnosis was supported by the temporal relationship with herbal product intake, a predominantly hepatocellular pattern, exclusion of viral and obstructive causes, absence of convincing evidence for autoimmune hepatitis, compatible liver biopsy findings, and progressive biochemical improvement after discontinuation of the suspected agent. This diagnostic approach is consistent with current DILI guidance, which emphasizes chronology, biochemical phenotype, exclusion of competing etiologies, and clinical course after withdrawal of the suspected product [1-3].

The initial biochemical profile was strongly hepatocellular. The marked elevation of ALT and AST, together with severe hyperbilirubinemia and impaired coagulation, indicated clinically significant liver injury. Using the conventional R-ratio approach, the patient’s initial values were compatible with a hepatocellular pattern. The presence of jaundice and coagulopathy warranted close monitoring. However, the absence of documented overt encephalopathy and the subsequent improvement in bilirubin, aminotransferases, and INR argued against progression to established acute liver failure. The favorable clinical and biochemical course after withdrawal of *Chelidonium majus* represents an important dechallenge response, strengthening the causal interpretation [1-4].

The differential diagnosis was systematically addressed. Viral hepatitis was excluded through negative testing for hepatotropic viruses, while magnetic resonance imaging/magnetic resonance cholangiopancreatography and ultrasonography showed no biliary dilatation, choledocholithiasis, or obstructive hepatobiliary lesion. Autoimmune hepatitis was also considered because of antinuclear antibody positivity and mildly elevated immunoglobulin G; however, autoantibody positivity may occur in DILI/HILI and is not diagnostic on its own [3, 4]. In this patient, anti-mitochondrial, anti-smooth muscle, and liver cytosol antibodies were negative, and liver biopsy did not show the typical plasma cell-rich interface hepatitis expected in classical autoimmune hepatitis. Instead, the histological pattern favored acute hepatitis-like toxic injury, with centrilobular necrosis, apoptotic hepatocytes, acidophil bodies, ceroid-laden macrophages, mild ductular reaction, and mild non-bridging fibrosis.

The liver biopsy was particularly useful because it supported the diagnosis and helped exclude alternative liver diseases. Preserved hepatic architecture, absence of steatosis, absence of significant cholestasis, absence of iron or alpha-1 antitrypsin accumulation, and lack of malignancy argued against several chronic, metabolic, storage, or structural causes. The observed lobular injury with centrilobular necrosis and apoptotic hepatocytes was compatible with an acute hepatitis-like pattern, which may be seen in DILI and HILI. Although histology alone cannot establish causality, in this clinical context, it provided strong supportive evidence for HILI.

The hepatotoxic potential of *Chelidonium majus* has been described for more than two decades. Benninger et al. reported ten cases of acute hepatitis associated with *Chelidonium majus* preparations, with variable severity and marked cholestasis in some patients, although liver failure was not observed [5]. Moro et al. subsequently reviewed the literature and reported an additional case, further supporting the association between *Chelidonium majus* intake and acute liver injury [6]. Teschke and colleagues later performed liver-specific causality evaluations of published European cases and spontaneous reports, emphasizing both the plausibility of *Chelidonium majus* hepatotoxicity and the importance of structured causality assessment [7,11]. Additional reviews and case reports have continued to identify *Chelidonium majus* as a potential cause of clinically relevant liver injury, including cases presenting with marked hypertransaminasemia and jaundice [8-10,12].

The pathophysiological mechanism of *Chelidonium majus* hepatotoxicity remains incompletely defined. The plant contains several biologically active alkaloids, including chelidonine, chelerythrine, sanguinarine, berberine, and coptisine, which may contribute to both pharmacological and toxicological effects [13]. However, available clinical data suggest an idiosyncratic pattern rather than predictable dose-dependent toxicity. This explains why causality assessment cannot rely only on exposure history. The updated RUCAM provides a structured framework for evaluating time to onset, post-withdrawal course, risk factors, concomitant agents, exclusion of alternative causes, prior hepatotoxicity information, and, when available, response to re-exposure [4]. In the present case, rechallenge was not performed and would not have been ethically appropriate, as recurrent exposure may provoke more severe liver injury.

This case is also clinically important in the broader context of herbal and dietary supplement-induced liver injury. Registry and review data show that herbal and dietary supplements represent an increasingly recognized cause of DILI and may occasionally be associated with severe outcomes, including acute liver failure [14-17]. Patients often perceive herbal products as harmless and may not report them unless specifically asked. Therefore, in any patient with unexplained acute hepatitis, the medication history should explicitly include non-prescribed products, herbal preparations, teas, extracts, weight-loss supplements, lipid-lowering natural products, and “liver detox” formulations. This is particularly relevant because herbal preparations may vary in composition, concentration, extraction method, purity, and potential for contamination or adulteration, complicating the assessment of causality [16,17].

Management of suspected *Chelidonium majus*-induced liver injury is mainly supportive. Immediate discontinuation of the suspected herbal product is essential, as no specific antidote exists. Monitoring should focus on the bilirubin trajectory, aminotransferase decline, coagulation parameters, and signs of hepatic encephalopathy. In the present case, conservative management with hydration, thiamine, and B-complex/multivitamin supplementation, vitamin K for impaired coagulation, and rifaximin/lactulose for hyperammonemia was followed by clinical and biochemical improvement. The initial presence of jaundice and coagulopathy justified inpatient monitoring, while the subsequent decline in bilirubin and INR supported recovery after withdrawal of the suspected agent [1-3].

The transient elastography result should be interpreted cautiously. Although the liver stiffness value was numerically high, it can overestimate fibrosis in acute hepatic inflammation [18,19]. In this patient, the elastography report itself noted that reliable staging was limited by inflammatory activity, and liver biopsy showed only mild non-bridging fibrosis with preserved architecture. Therefore, the stiffness measurement should not be interpreted as evidence of established cirrhosis.

## Conclusions

This case highlights *Chelidonium majus *as a clinically relevant cause of HILI. The patient’s presentation with jaundice, nausea, anorexia, and fatigue, followed by documented severe hepatocellular injury, hyperbilirubinemia, impaired coagulation, negative viral and obstructive work-up, compatible liver biopsy, and improvement after withdrawal of the herbal product, supports the diagnosis of probable *Chelidonium majus*-induced liver injury.

The case reinforces three important clinical messages. First, herbal products should be treated as potential hepatotoxic exposures, not as inherently safe alternatives to conventional drugs. Second, the diagnostic evaluation of acute hepatitis must include a detailed and explicit history of herbal medicines, dietary supplements, and non-prescribed preparations. Third, early recognition, discontinuation of the suspected agent, and close monitoring of hepatic synthetic function are essential, particularly when jaundice and coagulopathy are present.
